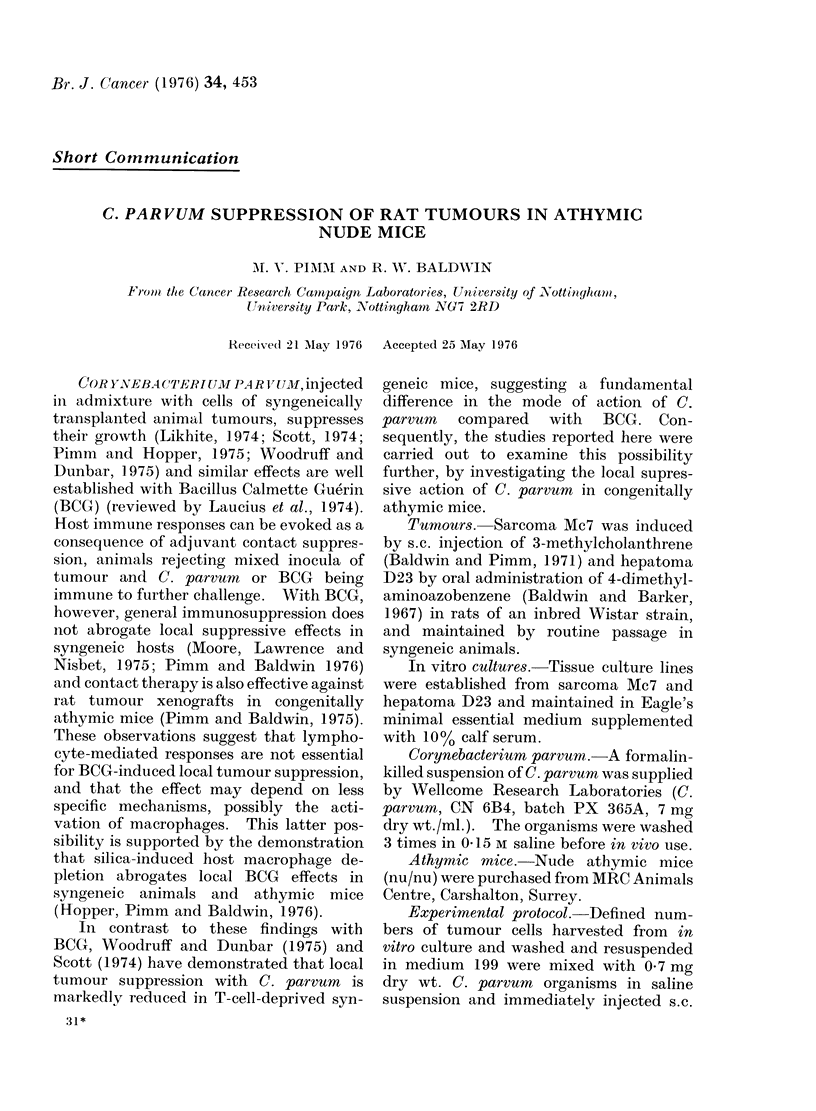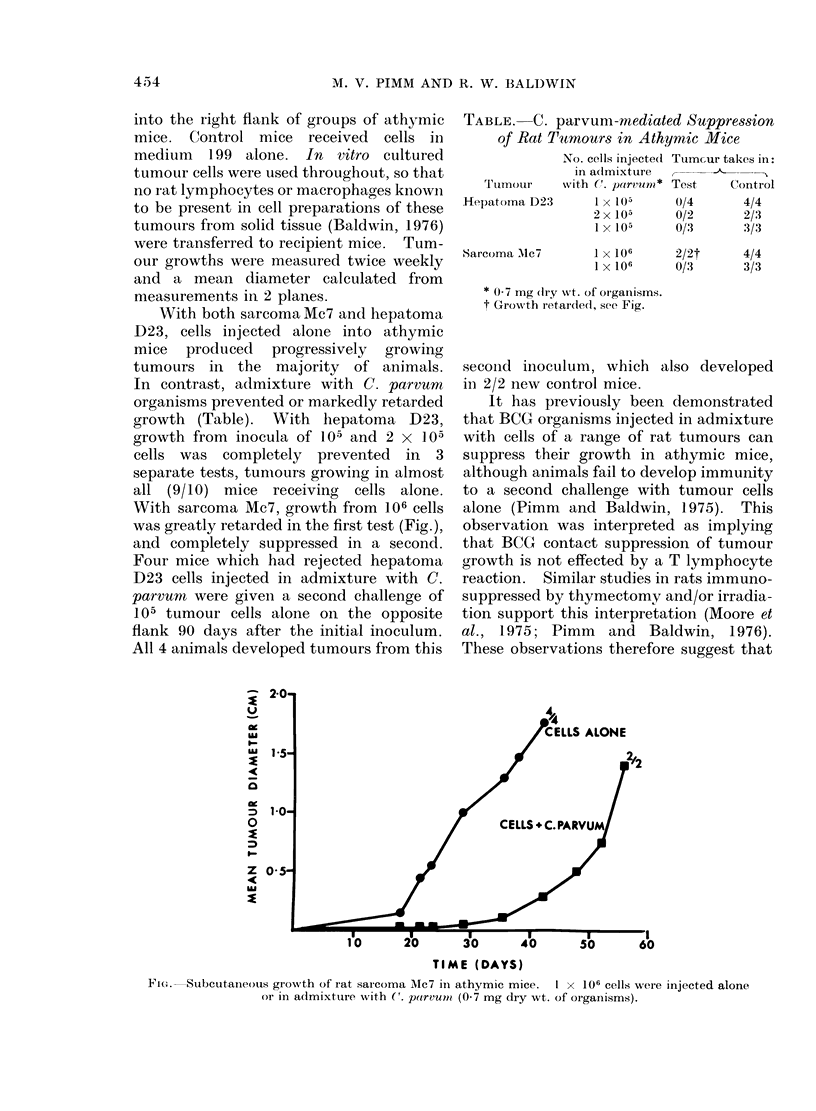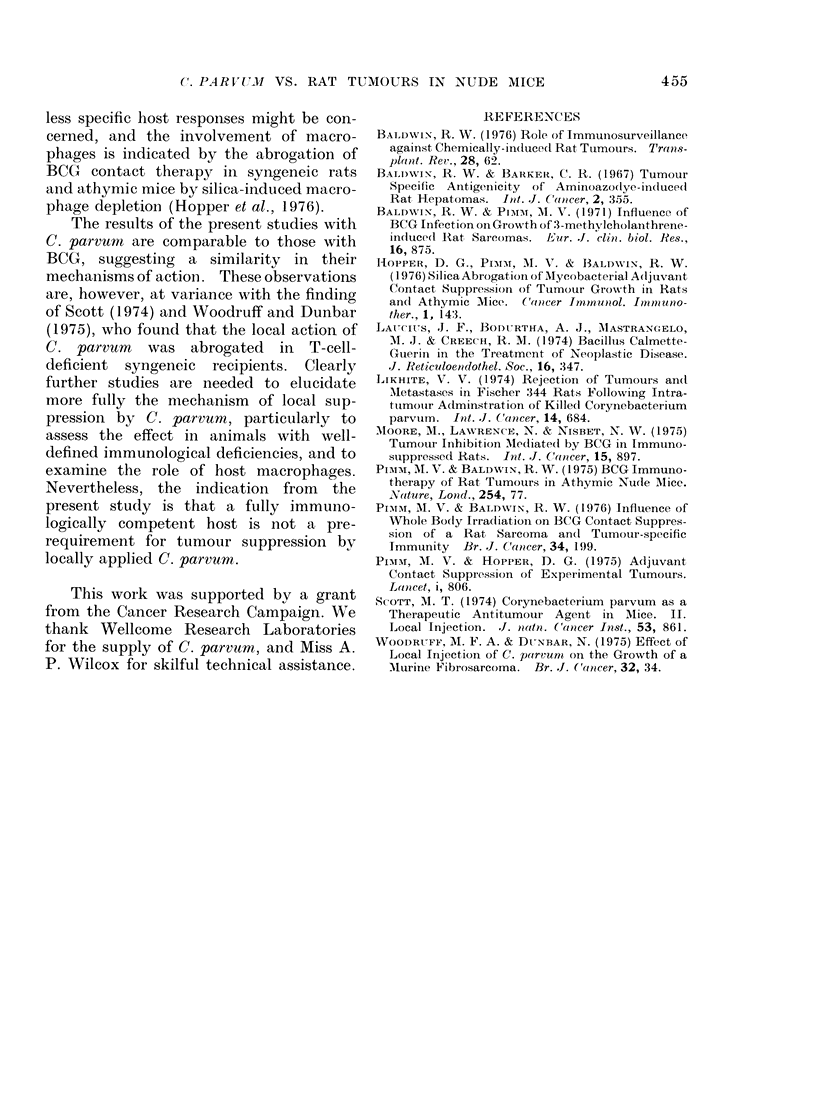# C. parvum suppression of rat tumours in athymic nude mice.

**DOI:** 10.1038/bjc.1976.191

**Published:** 1976-10

**Authors:** M. V. Pimm, R. W. Baldwin


Br. J. Cancer (1976) 34, 453

### Short Communication

### C. PARVUM SUPPRESSION OF RAT TUMOURS IN ATHYMIC NUDE MICE

M. V. PIMM AND R. W. BALDWIN

*From the Cancer Research Campaign Laboratories, University of Nottingham, University Park, Nottingham NG7 2RD*

Received 21 May 1976 Accepted 25 May 1976

CORYNEBACTERIUM PARVUM, injected in admixture with cells of syngeneically transplanted animal tumours, suppresses their growth (Likhite, 1974; Scott, 1974; Pimm and Hopper, 1975; Woodruff and Dunbar, 1975) and similar effects are well established with Bacillus Calmette Guérin (BCG) (reviewed by Laucius *et al.*, 1974). Host immune responses can be evoked as a consequence of adjuvant contact suppression, animals rejecting mixed inocula of tumour and *C. parvum* or BCG being immune to further challenge. With BCG, however, general immunosuppression does not abrogate local suppressive effects in syngeneic hosts (Moore, Lawrence and Nisbet, 1975; Pimm and Baldwin 1976) and contact therapy is also effective against rat tumour xenografts in congenitally athymic mice (Pimm and Baldwin, 1975). These observations suggest that lymphocyte-mediated responses are not essential for BCG-induced local tumour suppression, and that the effect may depend on less specific mechanisms, possibly the activation of macrophages. This latter possibility is supported by the demonstration that silica-induced host macrophage depletion abrogates local BCG effects in syngeneic animals and athymic mice (Hopper, Pimm and Baldwin, 1976).

In contrast to these findings with BCG, Woodruff and Dunbar (1975) and Scott (1974) have demonstrated that local tumour suppression with *C. parvum* is markedly reduced in T-cell-deprived syngeneic mice, suggesting a fundamental difference in the mode of action of *C. parvum* compared with BCG. Consequently, the studies reported here were carried out to examine this possibility further, by investigating the local supressive action of *C. parvum* in congenitally athymic mice.

*Tumours.*—Sarcoma Mc7 was induced by s.c. injection of 3-methylcholanthrene (Baldwin and Pimm, 1971) and hepatoma D23 by oral administration of 4-dimethylaminoazobenzene (Baldwin and Barker, 1967) in rats of an inbred Wistar strain, and maintained by routine passage in syngeneic animals.

In vitro *cultures.*—Tissue culture lines were established from sarcoma Mc7 and hepatoma D23 and maintained in Eagle's minimal essential medium supplemented with 10% calf serum.

*Corynebacterium parvum.*—A formalin-killed suspension of *C. parvum* was supplied by Wellcome Research Laboratories (*C. parvum*, CN 6B4, batch PX 365A, 7 mg dry wt./ml.). The organisms were washed 3 times in 0·15 M saline before *in vivo* use.

*Athymic mice.*—Nude athymic mice (nu/nu) were purchased from MRC Animals Centre, Carshalton, Surrey.

*Experimental protocol.*—Defined numbers of tumour cells harvested from *in vitro* culture and washed and resuspended in medium 199 were mixed with 0·7 mg dry wt. *C. parvum* organisms in saline suspension and immediately injected s.c.

into the right flank of groups of athymic mice. Control mice received cells in medium 199 alone. *In vitro* cultured tumour cells were used throughout, so that no rat lymphocytes or macrophages known to be present in cell preparations of these tumours from solid tissue (Baldwin, 1976) were transferred to recipient mice. Tumour growths were measured twice weekly and a mean diameter calculated from measurements in 2 planes.

With both sarcoma Mc7 and hepatoma D23, cells injected alone into athymic mice produced progressively growing tumours in the majority of animals. In contrast, admixture with *C. parvum* organisms prevented or markedly retarded growth (Table). With hepatoma D23, growth from inocula of $10^5$ and $2 \times 10^5$ cells was completely prevented in 3 separate tests, tumours growing in almost all (9/10) mice receiving cells alone. With sarcoma Mc7, growth from $10^6$ cells was greatly retarded in the first test (Fig.), and completely suppressed in a second. Four mice which had rejected hepatoma D23 cells injected in admixture with *C. parvum* were given a second challenge of $10^5$ tumour cells alone on the opposite flank 90 days after the initial inoculum. All 4 animals developed tumours from this second inoculum, which also developed in 2/2 new control mice.

TABLE.—C. parvum-*mediated Suppression of Rat Tumours in Athymic Mice*

| Tumour | No. cells injected in admixture with *C. parvum** | Tumour takes in: Test | Control |
|---|---|---|---|
| Hepatoma D23 | $1 \times 10^5$ | 0/4 | 4/4 |
| | $2 \times 10^5$ | 0/2 | 2/3 |
| | $1 \times 10^5$ | 0/3 | 3/3 |
| Sarcoma Mc7 | $1 \times 10^6$ | 2/2† | 4/4 |
| | $1 \times 10^6$ | 0/3 | 3/3 |

* 0·7 mg dry wt. of organisms.

† Growth retarded, see Fig.

It has previously been demonstrated that BCG organisms injected in admixture with cells of a range of rat tumours can suppress their growth in athymic mice, although animals fail to develop immunity to a second challenge with tumour cells alone (Pimm and Baldwin, 1975). This observation was interpreted as implying that BCG contact suppression of tumour growth is not effected by a T lymphocyte reaction. Similar studies in rats immunosuppressed by thymectomy and/or irradiation support this interpretation (Moore *et al.*, 1975; Pimm and Baldwin, 1976). These observations therefore suggest that

FIG.—Subcutaneous growth of rat sarcoma Mc7 in athymic mice. $1 \times 10^6$ cells were injected alone or in admixture with *C. parvum* (0·7 mg dry wt. of organisms).

less specific host responses might be concerned, and the involvement of macrophages is indicated by the abrogation of BCG contact therapy in syngeneic rats and athymic mice by silica-induced macrophage depletion (Hopper *et al.*, 1976).

The results of the present studies with *C. parvum* are comparable to those with BCG, suggesting a similarity in their mechanisms of action. These observations are, however, at variance with the finding of Scott (1974) and Woodruff and Dunbar (1975), who found that the local action of *C. parvum* was abrogated in T-cell-deficient syngeneic recipients. Clearly further studies are needed to elucidate more fully the mechanism of local suppression by *C. parvum*, particularly to assess the effect in animals with well-defined immunological deficiencies, and to examine the role of host macrophages. Nevertheless, the indication from the present study is that a fully immunologically competent host is not a prerequirement for tumour suppression by locally applied *C. parvum.*

This work was supported by a grant from the Cancer Research Campaign. We thank Wellcome Research Laboratories for the supply of *C. parvum*, and Miss A. P. Wilcox for skilful technical assistance.

### REFERENCES

BALDWIN, R. W. (1976) Role of Immunosurveillance against Chemically-induced Rat Tumours. *Transplant. Rev.*, **28**, 62.

BALDWIN, R. W. & BARKER, C. R. (1967) Tumour Specific Antigenicity of Aminoazodye-induced Rat Hepatomas. *Int. J. Cancer*, **2**, 355.

BALDWIN, R. W. & PIMM, M. V. (1971) Influence of BCG Infection on Growth of 3-methylcholanthrene-induced Rat Sarcomas. *Eur. J. clin. biol. Res.*, **16**, 875.

HOPPER, D. G., PIMM, M. V. & BALDWIN, R. W. (1976) Silica Abrogation of Mycobacterial Adjuvant Contact Suppression of Tumour Growth in Rats and Athymic Mice. *Cancer Immunol. Immunother.*, **1**, 143.

LAUCIUS, J. F., BODURTHA, A. J., MASTRANGELO, M. J. & CREECH, R. M. (1974) Bacillus Calmette-Guerin in the Treatment of Neoplastic Disease. *J. Reticuloendothel. Soc.*, **16**, 347.

LIKHITE, V. V. (1974) Rejection of Tumours and Metastases in Fischer 344 Rats Following Intratumour Adminstration of Killed Corynebacterium parvum. *Int. J. Cancer*, **14**, 684.

MOORE, M., LAWRENCE, N. & NISBET, N. W. (1975) Tumour Inhibition Mediated by BCG in Immunosuppressed Rats. *Int. J. Cancer*, **15**, 897.

PIMM, M. V. & BALDWIN, R. W. (1975) BCG Immunotherapy of Rat Tumours in Athymic Nude Mice. *Nature, Lond.*, **254**, 77.

PIMM, M. V. & BALDWIN, R. W. (1976) Influence of Whole Body Irradiation on BCG Contact Suppression of a Rat Sarcoma and Tumour-specific Immunity *Br. J. Cancer*, **34**, 199.

PIMM, M. V. & HOPPER, D. G. (1975) Adjuvant Contact Suppression of Experimental Tumours. *Lancet*, i, 806.

SCOTT, M. T. (1974) Corynebacterium parvum as a Therapeutic Antitumour Agent in Mice. II. Local Injection. *J. natn. Cancer Inst.*, **53**, 861.

WOODRUFF, M. F. A. & DUNBAR, N. (1975) Effect of Local Injection of *C. parvum* on the Growth of a Murine Fibrosarcoma. *Br. J. Cancer*, **32**, 34.